# Editorial: Greenhouse gas emissions and mitigation: microbes, mechanisms and modeling

**DOI:** 10.3389/fmicb.2024.1363814

**Published:** 2024-01-18

**Authors:** Baoli Zhu, Zengming Chen, Hangwei Hu, Adrain-Stefan Andrei, Yong Li

**Affiliations:** ^1^Key Laboratory of Agro-Ecological Processes in Subtropical Region, Institute of Subtropical Agriculture, Chinese Academy of Sciences (CAS), Changsha, China; ^2^Institute of Soil Science, Chinese Academy of Sciences (CAS), Nanjing, China; ^3^School of Agriculture, The University of Melbourne, Melbourne, VIC, Australia; ^4^Department of Plant and Microbial Biology, University of Zurich, Zurich, Switzerland; ^5^College of Environmental and Resource Sciences, Zhejiang University, Hangzhou, China

**Keywords:** greenhouse gas (CH_4_ N_2_O, CO_2_), climate change, nitrification, denitrification, ammonia oxidizing microorganisms

The soil, holding ~1500 Pg of total carbon (C) and 136 Pg of total nitrogen (N), represents the largest terrestrial reservoirs of these elements (Nieder and Benbi, 2008). Yet, it also stands as a significant source of greenhouse gas (GHG) emissions, contributing over 350 Pg CO_2_-equivalents annually and thereby significantly impacting global warming. Over the years, atmospheric N_2_O concentrations have risen by more than 20%, and CH_4_ concentrations have nearly tripled to 1900 ppb, primarily attributed to microbial activities (Schaefer et al., 2016). Understanding the microbial mechanisms alongside the production and reduction of GHGs is crucial. Recent discoveries, such as atypical nitrous oxide reductase (NosZ II), comammox, and novel processes like oxygenic denitrification and anaerobic oxidation of CH_4_ linked to the reduction of nitrate, nitrite, iron, and manganese oxides, underscore the pivotal role of soil microbes in regulating the biogeochemical cycles of C and N, and highlight avenues for targeted strategies to reduce GHG emissions and mitigate global warming. This Research Topic comprises nine articles that offer insights on the factors that influencing the emission of GHGs, especially N_2_O, and the potential roles of microorganisms.

Nitrification and denitrification are the main processes producing N_2_O. Fertilizer applications, especially N-fertilizers, fuel the emission of this potent greenhouse gas. Thus, nitrification inhibition can be a potential approach to reduce N_2_O emissions. In this Research Topic, Lei et al. analyzed over 200 datasets from 48 studies and found that application of nitrification inhibitors on average reduced about 60% of total N_2_O emission, increased over 70% of soil ammonium concentration, and decreased about 50% of AOB abundances. The findings emphasize AOB's significant role in N_2_O emissions, and can be a better indicator and target for N_2_O mitigation. Xie et al. compared N_2_O emissions from grasslands featuring a tropical grass species *Brachiaria humidicola*, whose root exudates with the capacity of biological nitrification inhibition, and a native grass *Eremochloa ophiuroide*, in Hainan, China. Interestingly, the N_2_O emission rate of the *B. humidicola* grassland was significantly higher, especially under N-fertilization treatment. Nevertheless, its yield-scaled N_2_O emission was significantly lower than the native *E. ophiuroide* grassland.

Nitrogen fertilizer application is also critical in influencing soil organic carbon (SOC) stability. Song et al. reported that nitrate addition enhanced the abundance and activity of SOC decomposers, thus, stimulating SOC decomposition in deep soils (>1 m), particularly when nitrate presented as the dominant electron acceptor over oxygen. This suggests the link between above-ground anthropogenic N input and deep soil carbon dynamics. Xu et al. demonstrated that delayed N fertilizer application in pea and maize intercropping reduced soil respiration rates and altered soil microbial community structures, thereby decreasing carbon emissions. This shed lights on agriculture management strategies in achieving carbon neutrality goals.

In addition, moisture plays important role in influencing greenhouse gas emissions and soil organic carbon (SOC) stability. Through laboratory incubations and literature synthesis, Wang et al. quantified N_2_O emission rates from nitrification and denitrification under different soil moisture levels (40% to 120% WFPS, water-filled pore space), and found that N_2_O emitting rate peaked at 80%−95% WFPS, while the dominating process switched from nitrification to denitrification when moisture increased over about 60% WFPS. Moisture as a major driver controls the relative contribution of nitrification and denitrification to N_2_O emissions was evident from synthesized 80 groups of data.

Han et al. investigated the responses of total microbial community and ammonium oxidizing microbes to short-term moisture level changes and nitrogen fertilizer application in paddy soils. Moisture influenced the abundance and composition of total soil microbes, and nitrogen fertilizer reduced the connectivity and complexity of the total bacteria network. The community structures of ammonium-oxidizing-bacteria (AOB) and -archaea (AOA) were largely influenced by ammonium and nitrate, respectively, which play crucial roles in nitrification, indicating a differential response of these microbes.

Qu et al. investigated the respiration rates of different layer soils of the Loess Plateau, and underscored soil temperature and moisture as critical factors influencing soil respiration rates, suggesting a positive feedback loop amplifying global warming. Yang et al. investigated the impact of maize and rice straw biochar on N_2_O emissions during paddy soil freeze-thaw cycles via simulating microcosm incubations. Results showed that biochar application decreased 10% of AOB abundance and reduced about two thirds of the total N_2_O emissions, revealing the application potential of biochar in decreasing soil N_2_O emissions.

By employing ^15^N tracing and N_2_O isotopocule methods, Karlowsky et al. dissected the contribution of bacterial denitrification and nitrifier denitrification to N_2_O emissions in hydroponic tomato cultivation system. Results indicated that bacterial denitrification, nitrifier denitrification and coupled nitrification and denitrification all contributed to the N_2_O emissions in the system.

In essence, these studies collectively offer profound insights into microbial mechanisms governing GHG emissions, presenting avenues for targeted mitigation strategies. More comprehensive and large-scale investigations are necessary to understand the intricate microbial processes driving GHG emissions, including methane, and to devise effective approaches to combat climate change.

## Author contributions

BZ: Writing – original draft, Writing – review & editing. ZC: Writing – original draft, Writing – review & editing. HH: Writing – original draft, Writing – review & editing. A-SA: Writing – original draft, Writing – review & editing. YL: Writing – original draft, Writing – review & editing.
